# Polymer-Magnetic Composite Particles of Fe_3_O_4_/Poly(*o*-anisidine) and Their Suspension Characteristics under Applied Magnetic Fields

**DOI:** 10.3390/polym11020219

**Published:** 2019-01-28

**Authors:** Jin Hee Lee, Qi Lu, Jae Yun Lee, Hyoung Jin Choi

**Affiliations:** Department of Polymer Science and Engineering, Inha University, Incheon 22212, Korea; 315193@inha.ac.kr (J.H.L.); 22172314@inha.edu (Q.L.); neostar9@naver.com (J.Y.L.)

**Keywords:** Fe_3_O_4_, poly(*o*-anisidine), magnetic particle, magnetorheological, suspension

## Abstract

Fe_3_O_4_/poly(*o*-anisidine) (POA) magnetic composite nanoparticles with their core-shell structure were synthesized by chemical oxidation polymerization technique and adopted as a magneto-responsive magnetorheological (MR) material. The chemical structure and morphology of core-shell nanoparticles were identified by FT-IR, SEM, TEM, and elemental analyzer. Pycnometer and vibrating sample magnetometer showed that the magnetic saturation and density of the Fe_3_O_4_/POA particles were reduced by the POA shell coatings. The rheological properties of the MR suspension dispersed in a silicone oil at various magnetic field strengths were investigated using a rotating rheometer under a magnetic field. The resulting MR suspension showed a typical Newtonian fluid behavior in the absence of external stimuli. When an external magnetic field was applied, it formed a strong chain structure, acting like a solid with a yield stress. Further solid-like behaviors were observed from storage shear relaxation and viscoelastic tests. Finally, the Fe_3_O_4_/POA nanoparticles showed better dispersion stability than pure Fe_3_O_4_ nanoparticles with 50% improvement.

## 1. Introduction

Magnetorheological (MR) suspensions in which magneto-responsive particles are dispersed in media such as silicone oil, mineral oil and hydrocarbons are field-responsive smart materials, demonstrating reversible transformation of their rheological properties in an external magnetic field [1,2,3,4]. Their rheological behaviors can be finely tuned by adjusting the strength of the external magnetic field. Without an external field, MR suspensions behave like a Newtonian fluid. However, as soon as the external field is applied, magnetic dipole–dipole interactions between the particles become dominant and the particles are aligned in the direction of the external field, forming a chain and transforming to a solid-like state within milliseconds, similar to electrorheological (ER) fluids under an applied electric field [5]. When the external field is being removed again, it returns back to a liquid state, showing the characteristics of Newtonian fluid [6,7,8,9]. These properties have been used in a variety of industrial applications such as brakes, dampers, clutches, and shock absorbers due to their excellent mechanical properties with large yield stresses [10,11].

As for the MR materials, various types of core-shell nanoparticles have been widely used because of their synergistic effect between core and shell including suspension stability [4,12,13,14], while until now, the most widely used magnetic materials for MR suspensions are soft-magnetic carbonyl iron (CI) microspheres because of their controllable and superior magnetic properties and appropriate particle size [15,16,17]. However, despite the excellent merits of CI particles as MR materials, CI-based MR suspensions generally cause problems of settling and redistribution because of the high density of CI particles (density of around 7.91 g/cc), limiting their engineering applications [18,19,20]. To improve this drawback, magnetite (Fe_3_O_4_) particles with sufficient magnetic behavior and lower density (density: 4.32 g/cc) than CI have been applied. However, the problem of precipitation still remains, and there is also a problem that Fe^2+^ contained in Fe_3_O_4_ can be easily oxidized and corroded [21,22]. A solution to this problem is to coat the surface of Fe_3_O_4_ particles with polymers [23,24]. Meanwhile, among various polymers, when the conducting polymer is used in the form of a conductive shell-magnetic core, not only MR but also ER characteristics under an electric field can also be obtained [25,26].

In this study, we synthesized polymer-magnetic composite nanoparticles with a core-shell structure using Fe_3_O_4_ particles and poly(o-anisidine) (POA) possessing an excellent reactivity. The particle morphology and magnetic properties of the Fe_3_O_4_/POA were examined using scanning electron microscope (SEM), transmission electron microscopy, and a vibrating sample magnetometer (VSM). Rheological properties of MR suspensions were further measured using a rotational rheometer under an applied magnetic field.

## 2. Experimental

### 2.1. Materials and Sample Preparation

Spherical Fe_3_O_4_ magnetic beads being used as a core part of Fe_3_O_4_/POA particles were synthesized using a solvothermal process, in which initially 10.8 g of ferric chloride hexahydrate (Sigma-Aldrich, St. Louis, MO, USA) and 14.4 g of sodium acetate (Sigma-Aldrich, St. Louis, MO, USA) were dissolved in 200 mL of ethylene glycol (Sigma-Aldrich, St. Louis, MO, USA) with vigorous stirring for a sufficient period of time. The prepared solution was transferred to a Teflon-coated stainless steel autoclave, sealed, and heated through a heating device at 200 °C for 24 h [27]. The resulting Fe_3_O_4_ magnetic particles were washed several times with ethanol and distilled water. Then, 1 g of Fe_3_O_4_ particles was placed in a reactor containing 400 mL of 0.1 M HCl and ultrasonicated for 30 min using a sonication bath (Powersonic 410, 40 kHz, 500 W, Hwashin Tech., Seoul, Korea). The reactor was stirred for 10 h at 5 °C, and then the particles and solution were separated using an external magnet. Both 100 mL of ethanol and 1 mL of ortho-anisidine monomer (DC Chemical, Seoul, Korea) were added to the reactor, and then the solution was sonicated for 30 min and slowly stirred at 5 degrees in a nitrogen atmosphere. After holding for 8 h, 1.7 mL of 12 M HCl was added, and 250 mL of ammonium persulfate was also added dropwise for 30 min. The solution was reacted under nitrogen for 8 h. Finally, the resulting product was washed several times with a magnet using ethanol and distilled water, and then dried in a vacuum oven at 60 °C for 24 h.

On the other hand, for the preparation of MR suspension with a 10 vol % particle concentration, Fe_3_O_4_/POA core/shell particles were dispersed uniformly in 100 cS silicone oil (KF-96, Shin-Etsu, Nagoya, Japan, density: 0.975 g/cc) using both an ultrasonifier and vortex mixer.

### 2.2. Characterization

The densities of both pure Fe_3_O_4_ and POA-coated Fe_3_O_4_ particles were measured by a gas pycnometer (AccuPyc 1130, Micromeritics Instruments Corporation, Norcross, Georgia, USA), while morphology and elemental composition of the Fe_3_O_4_/POA were characterized by high-resolution scanning electron microscope (HR-SEM) (SU-8010, Hitachi, Tokyo, Japan) combined with an energy dispersive X-ray analyzer (EDS) (EX-250, HORIBA, Kyoto, Japan). The transmission electron microscope (CM200, Philips, Amsterdam, The Netherlands) was also used to observe coated core-shell structures. The particle size distribution of the Fe_3_O_4_/POA was measured using a dynamic light scattering apparatus (DLS) (ELS-8000, Otsuka, Japan). The chemical constituent of POA-coated Fe_3_O_4_ was prepared by using KBr powder and analyzed via a Fourier transform infrared spectrophotometer (FT-IR) (VERTEX 80v, Bruker, Kanagawa, Japan) from the wavenumber of 400 to 4000 cm^−1^. The magnetic measurement was further performed in a powder form of the sample at room temperature though a vibrating sample magnetometer (VSM) (DMS 1660, Microsense, Lowell, USA) with a maximum magnetic field of 1200 kA/m. On the other hand, the MR properties were measured using a parallel-plate geometry (PP 20, gap distance: 0.5 mm) to a rotating rheometer (MCR 300, Anton Paar, Stuttgart, Germany) connected to a magnetic field from 0 to 342 kA/m. Steady shear behavior of the MR suspension tests was conducted with changes in a shear rate between 0.03 and 200 (s^−1^) using the MR suspension based on Fe_3_O_4_/POA core-shell particles. The amplitude sweep test was tested at a constant angular frequency of 6.28 rad/s in a strain range of 0.001–100%. The measurements of storage and loss modulus were made at each frequency range of 1–200 rad/s. Turbiscan (Classic, MA2000, L’Union, France) was used to confirm the stability of the MR suspension by measuring the light transmission of the suspension as a function of time from 0 to 65 min.

## 3. Results and Discussion

### 3.1. Material Characteristics

**Figure 1:**
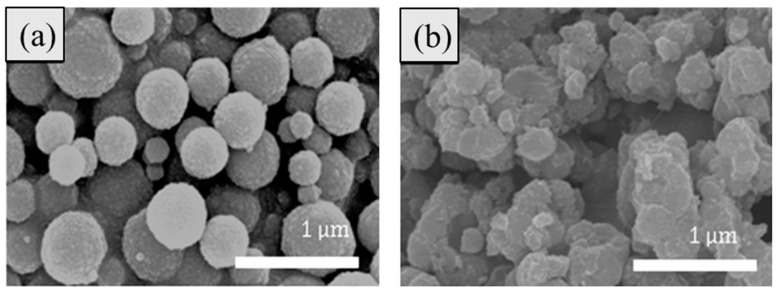
SEM images of (**a**) Fe_3_O_4_ spheres and (**b**) Fe_3_O_4_/POA core-shell spheres.

Figure 1 of the SEM image presents the morphology of spherical typed Fe_3_O_4_ and Fe_3_O_4_/POA particles, in which both particles have some polydispersed size distribution. While pure Fe_3_O_4_ in Figure 1a possesses a spherical shape with a rough surface, Figure 1b of the POA coated Fe_3_O_4_ core-shell particles showed a surface changed by POA on their surface after the polymer coating process. In addition, Table 1 and Table 2 show the EDS spectrum of both modified Fe_3_O_4_/POA and Fe_3_O_4_ particles, in which pure Fe_3_O_4_ represents the major peak of Fe 58.98% along with O 25.91% peak. Note that C peak of 15.11% also came from the carbon tape used. EDS data of POA-coated Fe_3_O_4_ particles were Fe 47.84%, O 22.43% peaks and C peak 29.74 %. It was observed that the carbon content increased from 15.11% to 29.74% by the coated polymeric shell layer. 

**Figure 2:**
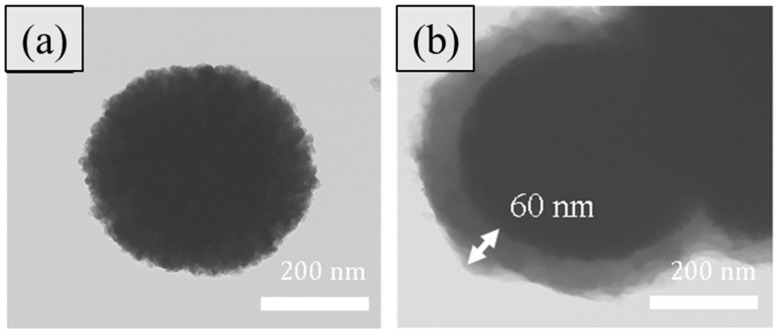
TEM images of (**a**) Fe_3_O_4_ spheres and (**b**) Fe_3_O_4_/POA core-shell spheres.

**Figure 3:**
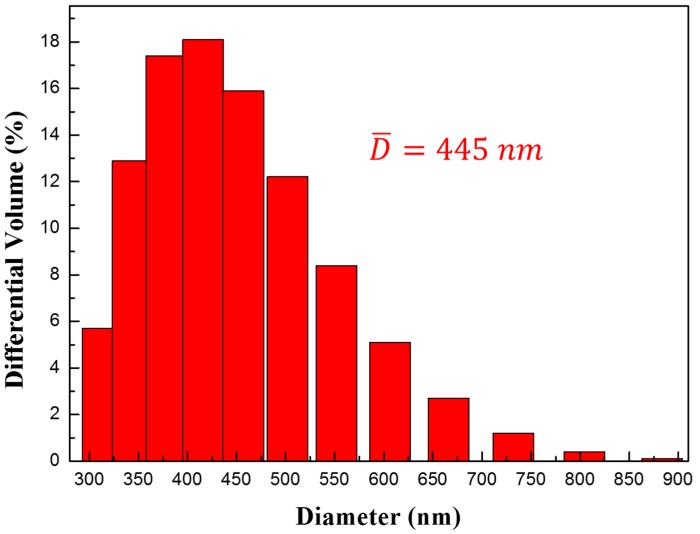
Size distribution of Fe_3_O_4_/POA particles.

Figure 2 displays TEM images of synthesized Fe_3_O_4_/POA core-shell nanoparticles. We can clearly find that the Fe_3_O_4_/POA nanoparticles are in the core-shell structure because after the acidification step of the Fe_3_O_4_ core, polymerization of the POA took place after the ortho-anisidine monomer was initially adsorbed on the Fe_3_O_4_ surface through the hydrogen bonding and electrostatic attraction. The Fe_3_O_4_ surface was modified only through the acidification process and no other surface treatment was required. The mean diameter of the Fe_3_O_4_ nanoparticles was about 300 nm and the average thickness of the polymeric POA shell was about 60 nm. This indicates that the surface of the Fe_3_O_4_ particles has been successfully coated with a conducting polymer. Furthermore, the Fe_3_O_4_/POA particles were dispersed in water and their particle size distribution was measured using a dynamic light scattering (DLS) apparatus as shown in Figure 3. It shows that the diameter of the particles varied from 310 to 890 nm and had an average value of 445 nm, while some agglomerated particles are observed from the TEM and SEM images because of their dried particle state during their tests.

**Figure 4:**
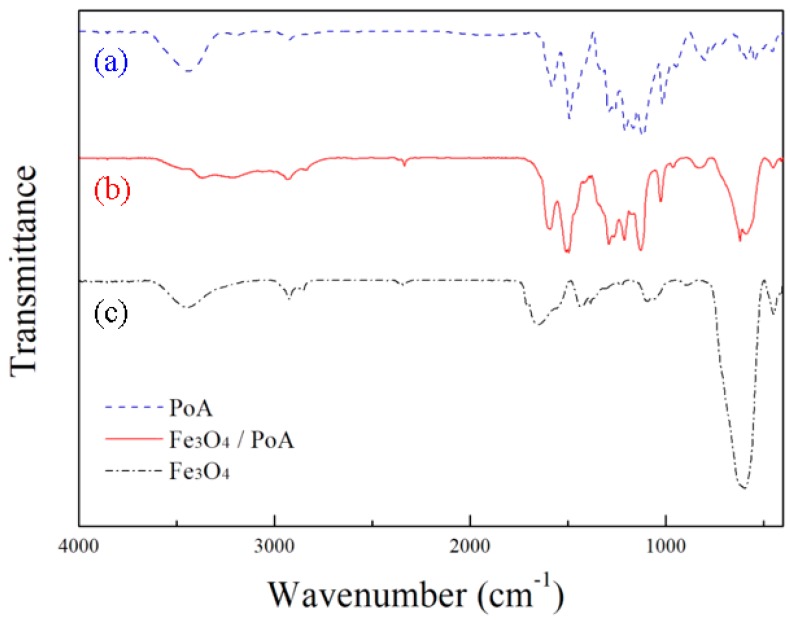
FT-IR absorption spectra of (a) POA, (b) Fe_3_O_4_/POA, and (c) Fe_3_O_4._

The chemical composition of Fe_3_O_4_/POA core-shell microspheres was confirmed further by FT-IR spectroscopy as shown in Figure 4. By a comparison of the FT-IR spectra of the Fe_3_O_4_ particles, neat POA and POA-coated Fe_3_O_4_ particles shown in Figure 4 illustrated the difference in the wavenumbers of the respective spectra. All samples were prepared using KBr in their pellet form. The stretching bands of POA in curve (a) of Figure 4 were 3416 cm^−1^, 1498 cm^−1^ from N–H, C=C benzenoid rings and 1580 cm^−1^ from C=C quinoid rings. The C-H in plane bending of the quinoid rings also appeared at 1116 cm^−1^. The curve (c) in Figure 4 showed an Fe-O stretching band at 586 cm^−1^. Both POA and Fe_3_O_4_ peaks are all shown in curve (b) of Figure 4, which is the analysis data of Fe_3_O_4_/POA particles.

**Figure 5:**
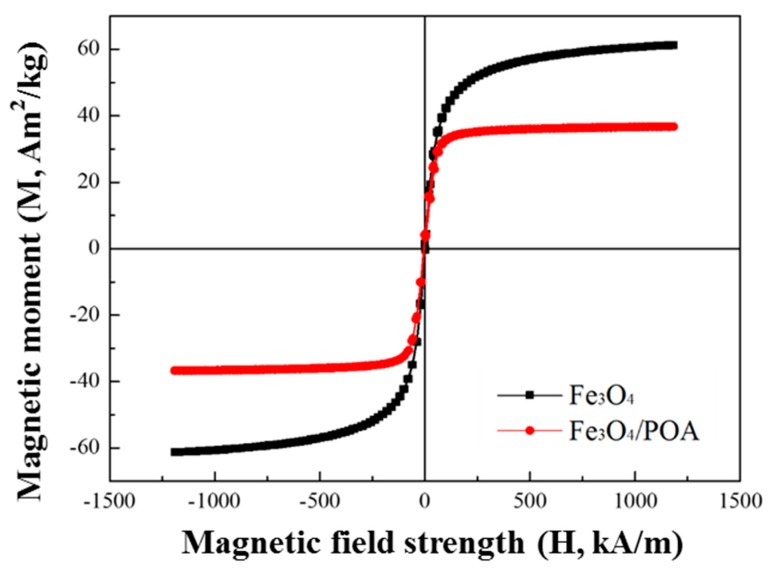
VSM curves of pure Fe_3_O_4_ particles and Fe_3_O_4_/POA core-shell microspheres.

On the other hand, Figure 5 shows the magnetic hysteresis curves of both Fe_3_O_4_ and Fe_3_O_4_/POA particles tested using the VSM, in which the magnetic moment was measured as a function of the magnetic field strength of −1200 kA/M at 1200 kA/m. The two different magnetic particles showed different magnetic moment values, particularly that the Fe_3_O_4_/POA particles had a magnetization close to the saturation state of 36 emu/g while the Fe_3_O_4_ powder had that of 61 emu/g. These values indicated that the magnetic properties of the magnetic particles coated with polymer decreased, and the density of Fe_3_O_4_/POA coated with conductive polymer POA decreased from 4.34 g/cm^3^ to 2.52 g/cm^3^. This reduction in saturation magnetization was due to the introduction of a non-magnetic POA-coated shell.

### 3.2. Magnetorheological Characteristics

**Figure 6:**
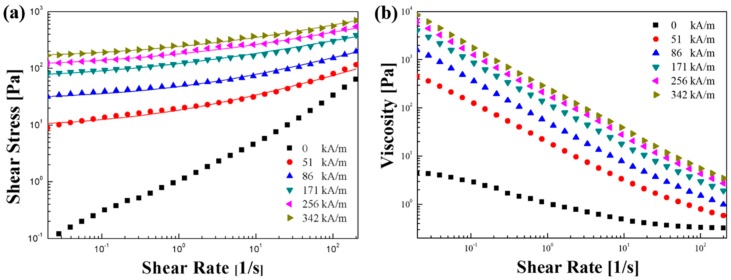
Flow curve for Fe_3_O_4_/POA (10 vol %) MR suspension under different magnetic field strengths. (**a**) Shear stress and (**b**) shear viscosity as a function of shear rate.

In order to study the MR characteristics of the Fe_3_O_4_/POA core/shell particles, a magnetic fluid of 10 vol % was prepared using silicone oil having a density of 0.965 g/cm^3^ (KF-96-100cSt, Shin-Etsu Silicone). The MR performance was measured using a rotational rheometer in a controlled shear rate (CSR) mode. Figure 6 shows the constant steady shear behavior of the MR suspension. Tests were conducted with changes in a shear rate between 0.01 and 200 (/s) using the MR suspension based on Fe_3_O_4_/POA core-shell particles. The measurement interval was set from the initial 10 s to the final 0.5 s through the log–log scale of each sweep test. The resulting flow curve response was investigated as a function of field strength in the range of 0 to 342 kA/m. Without the magnetic field, the shear stress and shear viscosity increases linearly associated with the Newtonian fluid behavior, whereas the magnetic field increases the shear stress and shear viscosity of the MR suspension. Following the Herschel–Bulkley model when a magnetic field is applied, a linear behavior with increasing slope in the sense of a high shear rate in the low shear rate range was observed in a relatively log–log plot. As expected, the shear stresses obtained as in previous studies using MR suspensions tend to be highly dependent on the applied field strength. Due to strong dipole interaction between adjacent core-shell magnetic particles, a solid column is formed and typical MR behavior is shown. As shown in Figure 6b, the shear thinning behavior was observed in MR fluid at a fixed magnetic field strength. For the shear viscosity of MR suspensions, the shear rate is reduced as the structure increases, and the characteristics of non-Newtonian fluids with shear losses due to structural deformation and fracture are shown [28,29].

The MR efficiency for evaluating the magnetic response performance of the MR suspension for different applied magnetic fields can be estimated using the following Equation (1);

(1) $$I\;=\;(\tau_{M}-\tau_{0})/\tau_{0}\;\times \;100(\%)\;\mathrm{or}\;I\;=\;(\eta_{M}-\eta_{0})/\eta_{0}\;\times \;100(\%)$$

**Figure 7:**
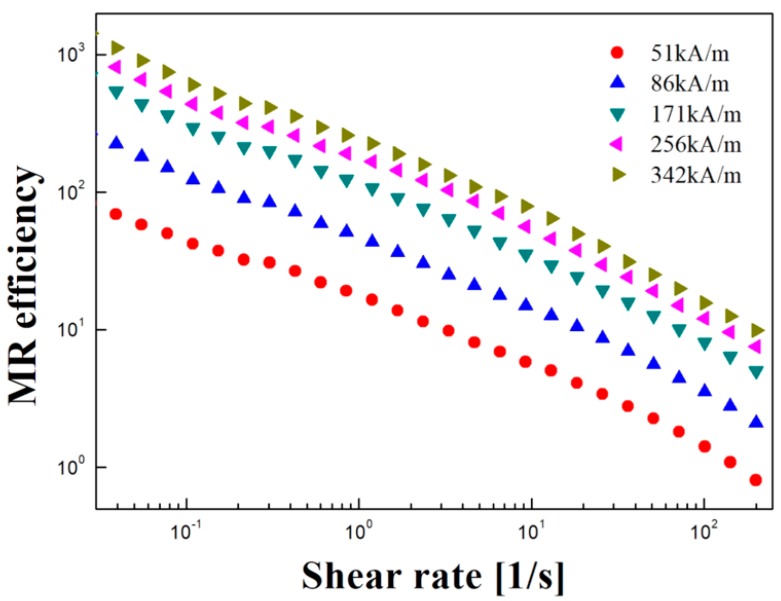
MR efficiency of Fe_3_O_4_/POA based MR suspension.

In Equation (1), *τ*_0_ and *η*_0_ are shear stress and shear viscosity, respectively, in the absence of applied magnetic field, and *τ_M_* and *η_M_* are shear stress and shear viscosity under applied magnetic field [30]. As shown in Figure 7, the relative value of magnetic efficiency increases with increasing magnetic field strength, and it decreases sharply with an increasing shear rate when the magnetic field is applied. This indicates the gradual destruction of the internal structure of the MR suspension that was forming the chain by the magnetic field when an external stimulus was given. For the controlled shear rate mode data, the shear stress results were analyzed using a commonly used rheological model of a Herschel–Bulkley rheological equation of state of Equation (2) for each magnetic field;

(2) $$\tau \;=\;\tau_{y}\;+\;K\;{\dot{\gamma }}^{n}$$

where *τ_y_* is the yield stress, *K* is the consistency, and n is the flow behavior index. The results obtained from the different magnetic fields can be used to determine the evolution of the parameters with respect to the applied magnetic field using the proposed magnetization model [29]. 

**Figure 8:**
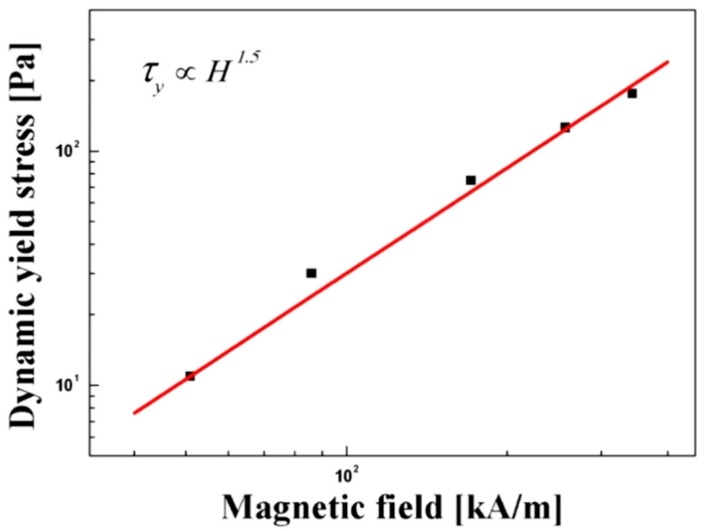
Dynamic yield stress as a function of magnetic field.

In Figure 8, the dynamic yield stress which was obtained by extrapolating shear stress to zero shear rate was reanalyzed as a function of the magnetic field strength for the Fe_3_O_4_/POA-based MR suspension. When there are external stimuli such as magnetic field (*H*), the plot shows the exponential relationship between dynamic yield stress and *H* as given in Equation (3);

(3) $$\tau_{y}\propto H^{\alpha }$$

In general, the power law index was proposed to be 1.5 for the intermediate case and 2.0 for the magnetic polarization model. The Fe_3_O_4_/POA MR suspension has a slope of 1.5 for the MR system, probably its weakened dependence due to non-magnetic POA association.

**Figure 9:**
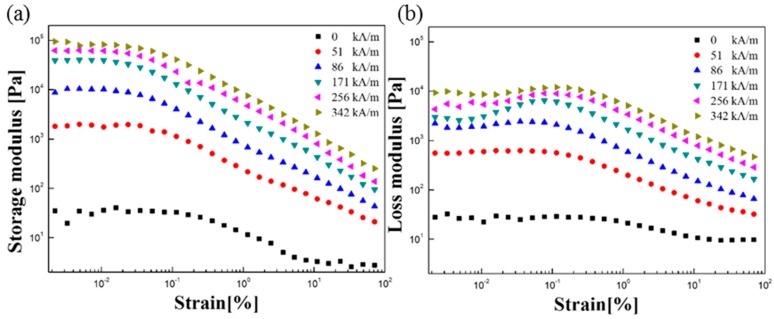
Amplitude sweep dependence of (**a**) storage modulus (G′) and (**b**) loss modulus (G″) for Fe_3_O_4_/POA-based MR suspension.

The amplitude sweep test was performed initially to determine the linear viscoelastic region (LVE) at a constant angular frequency of 6.28 rad/s in a strain range of 0.001–100%. Figure 9a,b shows the storage modulus (G′) and loss modulus (G″) as a function of shear strain with a magnetic field. In the low strain region, both moduli were independent of the strain in the LVE region, and a specific strain (0.005%) was chosen for each strain sweep test. G′ and G″ decreased as a result of irreversible structural changes in the MR suspension when the applied strain was increased sufficiently above the plateau region. When the magnetic field is applied, the MR suspension forms a solid structure while forming a chain during a state change from a liquid state to a solid state. The values of storage modulus (G′) and loss modulus (G″) were measured by the frequency sweep test after defining the LVE area of 0.005% according to the results of the amplitude sweep test in Figure 9. 

**Figure 10:**
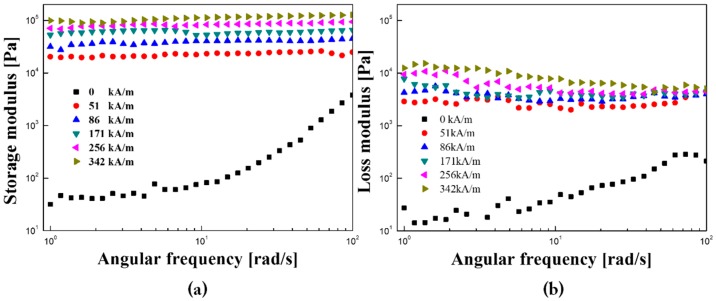
(**a**) Storage modulus; (**b**) loss modulus for core-shell microspheres Fe_3_O_4_/POA (10 vol %) MR suspension under various magnetic field strengths.

As shown in Figure 10, G′ and G″ were measured as a function of angular frequency as the field strength increased in constant strain. The measurements were made at each frequency range of 1–200 rad/s. When there is no magnetic field, G′ and G″ increase as the field strength increases, and the two values are very similar, indicating a gel-like state. The presence of a magnetic field showed that the storage modulus was higher than the loss factor and that solid-like behavior predominated over liquid-like behavior. Also, the constant value of the storage modulus shows that the chain structure of the Fe_3_O_4_/POA-based MR suspension is not destroyed in the respective frequency ranges measured, so that the viscoelastic fluid having vibration absorption ability to be maintained. In the various magnetic field ranges, G′ exhibited a broad plateau area under all different intensity fields and showed an increase in proportion to magnetic intensity. These results reflect the enhanced MR effect of the fluid through higher elasticity and strong MR behavior in the presence of the MR suspension in the presence of the magnetic field [31,32].

**Figure 11:**
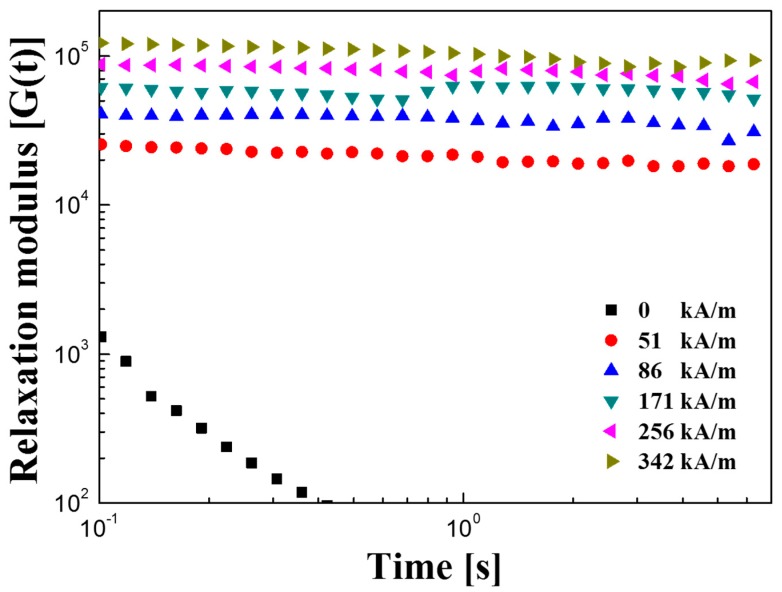
Shear relaxation modulus G(t) of Fe_3_O_4_/POA-based MR suspension.

Figure 11 shows a relaxation modulus as a function of time. The stress relaxation modulus G(t) is an important factor in the analysis of the phase change behavior of MR suspensions. The stress relaxation behavior is related to the phase transition from the liquid phase to the solid phase. The Schwarzl equation, represented by Equation (4), can be used to predict the relaxation behavior of the MR suspension using the measurements of G′(ω) and G″(ω) obtained from the frequency sweep test as follows;

(4) $$G\;(t)≅G^{\prime }\;(\omega )-0.566\;G^{\prime \prime }\;(\omega /2)+0.203\;G^{\prime \prime }\;(\omega )$$

As shown in Figure 10, when a magnetic field was applied, G(t) had a substantially constant value and increased in proportion to the intensity of the magnetic field. This indicates that the chain structure formed in response to the applied magnetic field behaves like a solid. When there is no magnetic field, G (t) decreases with time [33,34].

### 3.3. Sedimentation Characteristics

**Figure 12:**
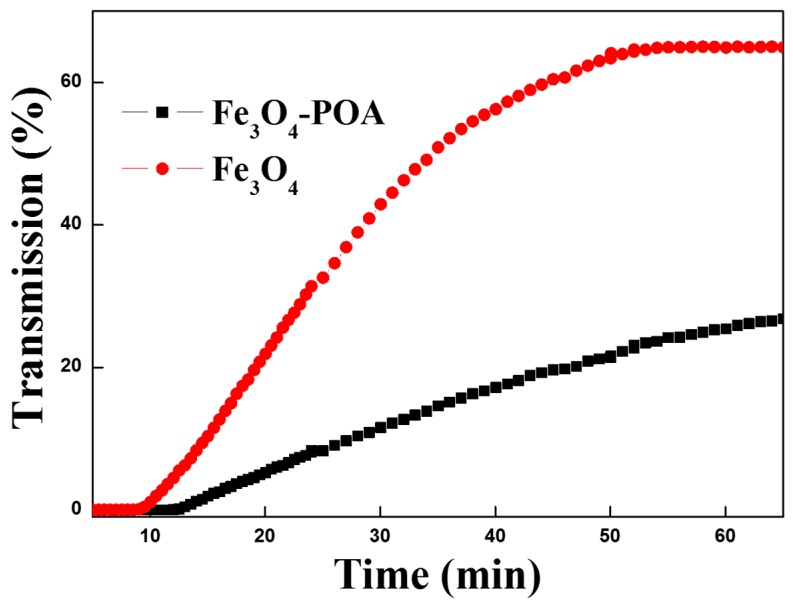
Transmission profiles of pure Fe_3_O_4_ and Fe_3_O_4_/POA MR suspension.

The dispersion stability of core-shell Fe_3_O_4_ and Fe_3_O_4_/POA particle MR suspension was measured using Turbiscan as shown in Figure 12. The degree of sedimentation was confirmed using the light transmittance over time. Because the Fe_3_O_4_ particles precipitate faster than the POA-coated Fe_3_O_4_ particles, the coated core-shell particles form a relatively more stable dispersion. Sedimentation of the Fe_3_O_4_/POA MR suspension was slowed down more than 50%. Through this, iron oxide particles coated with polymer with lower density have been proposed as a solution to the problem of precipitation of MR suspension [35].

## 4. Conclusions

A new type of polymer-magnetic composite Fe_3_O_4_/POA nanoparticles was prepared by coating magnetic particles with the conducting polymer to improve the precipitation stability of the magnetic particles, using Fe_3_O_4_ particles fabricated via a hydrothermal synthesis. The particle size, shell thickness and morphology were observed by SEM and TEM, and the chemical composition of the synthesized particles was confirmed by FT-IR. The rheological properties of the MR suspension in the presence of applied magnetic field strengths were analyzed through a rotational rheometer. The testing results showed that shear stress behavior with shear rate follows the Herschel–Bulkley model. The dynamic yield stress was fitted as the slope of 1.5. The G′ and G″ values and MR efficiency of the fluid increased with the magnetic field strength. The relaxation modulus as a function of time also showed the phase change behavior of MR suspensions from liquid-like to solid-like. In addition, the decreased particle density of Fe_3_O_4_/POA particles (density: 2.53 g/cc) to that of pure Fe_3_O_4_ (density: 4.32 g/cc) with a core-shell structure indicated their improved dispersion stability. This kind of MR suspension is expected to be applied, including automotive primary suspensions, truck seat systems, control-by-wire/tactile-feedback devices, pneumatic control, seismic mitigation, and human prosthetics, as the advantages of real-time control possibilities and dispersion stability.

## Figures and Tables

**Table 1:** EDAX data of Fe_3_O_4_ spheres.

Element	Weight%	Atomic%
C K	15.11	31.98
O K	25.91	41.17
Fe K	58.98	26.84
Total	100	

**Table 2:** EDAX data of Fe_3_O_4_/POA spheres.

Element	Weight%	Atomic%
C K	29.74	52.30
O K	22.43	29.61
Fe K	47.84	18.09
Total	100	
